# The complete mitochondrial genome of *Paracolopha morrisoni* (Baker, 1919) (Hemiptera: Aphididae)

**DOI:** 10.1080/23802359.2019.1666046

**Published:** 2019-09-18

**Authors:** Jieun Lee, Jonghyun Park, Hyobin Lee, Jongsun Park, Wonhoon Lee

**Affiliations:** a Department of Plant Medicine, Gyeongsang National University, Jinju, The Republic of Korea;; b InfoBoss Co., Ltd, Seoul, The Republic of Korea;; c InfoBoss Research Center, Seoul, The Republic of Korea;; d Institute of Agriculture & Life Science, Gyeongsang National University, Jinju, The Republic of Korea

**Keywords:** *Paracolopha morrisoni*, mitochondrial genome, Eriosomatinae, Aphididae, Korea

## Abstract

We have determined the mitochondrial genome of *Paracolopha morrisoni* (Baker, 1919), a gall-forming aphid collected from Korea. The circular mitogenome of *Paracolopha morrisoni* is 16,330 bp long including 13 protein-coding genes, 2 ribosomal RNA genes, 22 transfer RNAs, and a single large non-coding region of 932 bp. The base composition was AT-biased (84.9%). Gene order of *P. morrisoni* is identical to all other aphid mitochondrial genomes. Phylogenetic trees show that *P. morrisoni* is sister to *Eriosoma lanigerum* both belonging to tribe. The mitochondrial genome of *P. morrisoni* will be useful in understanding the genetic backgrounds of the species.

*Paracolopha morrisoni* is an aphid widely occurring in eastern Asian countries (China, Japan, and Korea), Unites States (Maryland, and South Carolina), and Europe (Belgium. Britain, Italy, and The Netherlands; Si 1985; Malumphy 2012). In Asia, *P. morrisoni* are known to be heteroecious, altering their hosts from a primary host *Zelkova serrata* to the secondary host, bamboo (*Sasa* spp.; Si 1985). Outside of Asia on the other hand, they are only found on roots of bamboo species, both introduced (*Phyllostachys* and *Pleioblastus* spp., native to China) and native species (*Arundinaria gigantea*, native to the US). Since *P. morrisoni*, in America and Europe, are mostly found in cultivated bamboos and do not alternate hosts, it has been believed these aphids originated in east Asia but were introduced overseas hitchhiking international trades of host plants. This is not completely confirmed, however, since the North American populations show novel characteristics such as alates occurring in spring (Si 1985). To understand genetic background of this species, we determined the complete mitochondrial genome of *P. morrisoni* collected from South Korea.

Total DNA of *P. morrisoni* was extracted from wingless females collected on *Zelkova serrata* from Haymang-gun, Gyeongsangnam-do, Korea in 2019 (35°54′20″N, 126°76′84″E; the specimen is stored in Gyeongsang National University, Korea accession number: Coll#JE147) using DNeasy Brood & Tissue Kit (QIAGEN, Hilden, Germany). Raw sequences obtained from Illumina HiSeqX at Macrogen Inc., Korea, were filtered by Trimmomatic 0.33 (Bolger et al. 2014) and *de novo* assembled and confirmed by Velvet 1.2.10 (Zerbino and Birney 2008), SOAPGapCloser 1.12 (Zhao et al. 2011), BWA 0.7.17 (Li et al. 2009), and SAMtools 1.9 (Li 2013). Geneious R11 11.1.5 (Biomatters Ltd., Auckland, New Zealand) was used for annotation based on that of *Eriosoma lanigerum* (NC_033352). ARWEN (Laslett and Canbäck 2008) was used to annotate tRNAs.

*Paracolopha morrisoni* mitochondrial genome length (Genbank accession is MN167467) is 16,330 bp and GC ratio is 15.1%, showing AT-biased. It contains 13 protein-coding genes, 2 rRNAs, and 22 tRNAs. The tRNAs size ranges from 53 to 73 bp, similar to other aphids (52–90 bp). Gene order of *P. morrisoni* is identical to that of all other aphid mitogenomes which are apparently the ancestral gene order of all insects (Wang et al. 2013).

All available complete mitochondrial genomes of 34 aphids including *P. morrisoni* and *Bembisia tabaci* (NC_006279) as an outgroup were aligned by MAFFT 7.388 (Katoh and Standley 2013). Bootstrapped maximum-likelihood (bootstrap repeat is 1000) and neighbor-joining (bootstrap repeat is 10,000) trees were constructed using MEGA X (Kumar et al. 2018). Phylogenetic trees show that all valid aphid tribes are monophyletic with *P. morrisoni* in Erisomatini (Figure 1). However, our tree topologies also presented Erisomatini grouped with Greenideidae resulting in paraphyletic manner of subfamily Eristomatinae, which is not the first time to be reported (Nováková et al. 2013). Our mitochondrial genome will be a key resource in understanding the genetic backgrounds and phylogenetic position of *P. morrisoni*.

**Figure 1.**
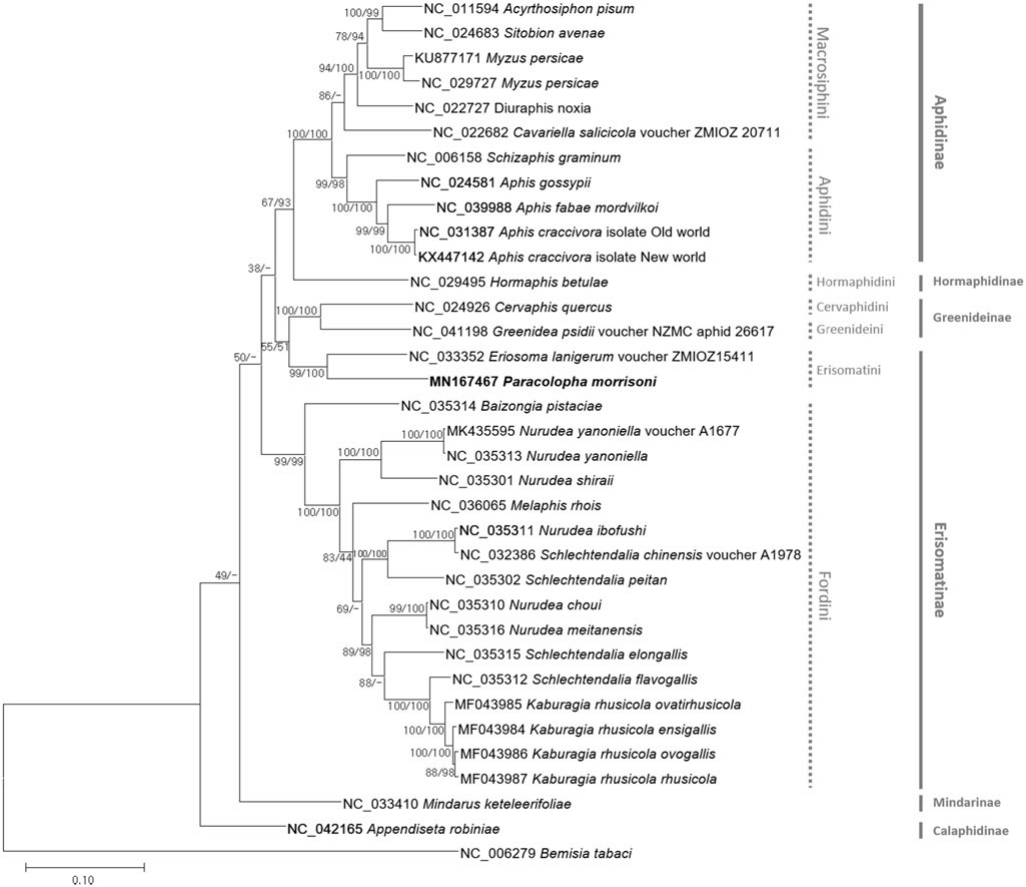
Maximum-likelihood (bootstrap repeat is 1000) and neighbor-joining (bootstrap repeat is 10,000) phylogenetic trees of 34 aphids and one whitefly mitochondrial genomes: *Paracolopha morrisoni* (MN167467, this study), *Acyrthosiphon pisum* (NC_011594), *Sitobion avenae* (NC_024683), *Myzus persicae* (NC_029727, KU877171), *Diuraphis noxia* (NC_022727), *Cavariella salicicola* (NC_022682), *Schizaphis graminum* (NC_006158), *Aphis gossypii*(NC_024581), *Aphis fabae mordvilkoi* (NC_039988), *Aphis craccivora* (NC_031387, KX447142), *Hormaphis betula* (NC_029495), *Cervaphis quercus* (NC_024926), *Greenidea psidii* (NC_041198), *Eriosoma lanigerum* (NC_033352), *Baizongia pistaciae* (NC_035314), *Nurudea yanoniella* (NC_035313, MK435595), *Nurudea shiraii* (NC_035301), *Melaphis rhois* (NC_036065), *Nurudea ibofushi* (NC_035311), *Schlechtendalia chinensis* (NC_032386), *Schlechtendalia peitan* (NC_035302), *Nurudea choui* (NC_035310), *Nurudea meitanensis* (NC_035316), *Schlechtendalia elongallis* (NC_035315), *Schlechtendalia flavogallis* (NC_035312), *Kaburagia rhusicola ovatirhusicola* (MF043985), *Kaburagia rhusicola ensigallis* (MF043984), *Kaburagia rhusicola ovagallis* (MF043986), *Kaburagia rhusicola rhusicola* (MF043987), *Mindarus keteleerifoliae* (NC_033410), *Appendiseta robiniae* (NC_042165), and *Bemisia tabaci* (NC_006279) as an outgroup. Phylogenetic tree was drawn based on the maximum-likelihood tree. The numbers above branches indicate bootstrap support values of maximum-likelihood and neighbor joining phylogenetic tree, respectively.
